# Factors of hospitalization expenditure of the genitourinary system diseases in the aged based on “System of Health Account 2011” and neural network model

**DOI:** 10.7189/jogh.08.020504

**Published:** 2018-12

**Authors:** Junlin He, Zhuo Yin, Wenjuan Duan, Yushan Wang, Xin Wang

**Affiliations:** 1 School of Public Health, China Medical University, Wuxi No. 2 People’s Hospital Affiliated to Nanjing Medical University, Wuxi, China; 2 College of Metropolitan Transportation, Beijing University of Technology, Beijing, China; 3 College of the Humanities and Social Sciences, China Medical University, Shenyang, China; 4 China Medical University, Shenyang, China; 5 College of the Humanities and Social Sciences, China Medical University, School of Public Health, Xinjiang Medical University, Urumqi, China

## Abstract

**Background:**

Hospitalization expenditure of genitourinary system diseases among the aged is often overlooked. The aim of our research is to analyze the basic situation and influencing factors of hospitalization expenditure of the genitourinary system diseases and provide better data for the health system.

**Methods:**

A total of 1 377 681 patients aged 65 years and over were collected with multistage stratified cluster random sampling in 252 medical institutions in Liaoning China, and “System of Health Account 2011” (SHA2011) was conducted to analyze the expenditure of the diseases. The corresponding samples were extracted, the neural network model was utilized to fit the regression model of the diseases among the aged, and sensitivity analysis was used to rank the influencing factors.

**Results:**

Total hospitalization expenditure in Liaoning was 51.286 billion yuan, and curative care expenditure of diseases of the genitourinary system was 3.350 billion yuan, accounting for 6.53%. In the neural network model, the training set of R2 was 0.71. The test set of R2 was 0.74. In the sensitivity analysis, top-three influencing factors were the length of stay, type of institutions and type of insurances; the weight was 0.28, 0.19 and 0.14, respectively.

**Conclusions:**

This research used SHA2011 to grab a large amount of data and analyzed them depending upon the corresponding dimensions. The neural network can analyze the influencing factors of hospitalization expenditure of genitourinary diseases in elderly patients accurately and directly, and can clearly describe the extent of its impact by combining sensitivity analysis.

Recently, the ageing population and the burden of disease have become the hottest public health issues in the world and have brought huge pressure to the health system in developed countries and many developing countries [1]. About 23% of the burden of disease was contributed by the elderly population aged 65 and over [2]. By the year of 2014, the population aged 65 and above in China accounted for 10.1% of total population, which was also the main population of chronic non-communicable diseases. Moreover, the incidence of the elderly population in chronic non-communicable disease rate is 2-3 times compared to the total population [3]. As a result, the cost of health care for the elderly population is much higher.

Diabetes and hypertension are the principal causes of chronic kidney disease (CKD) in all high-income and middle-income countries, and also in many developing areas [4]. The rapid increase in the prevalence of these risk factors will lead to a greater burden of CKD [5]. For example, people with CKD are five to ten times more likely to die prematurely than they are to progress to end-stage kidney disease. This increasing risk of death rises exponentially as kidney function worsens and is largely attributable to death from cardiovascular disease, although cancer incidence and mortality are further increased [6]. In conclusion, mortality and morbidity in high-risk individuals with diabetes and hypertension are often associated with the genitourinary system diseases related factors. At present, limited research and data on the aging population of hospitalization expenditure of genitourinary system diseases, and most studies focused on the epidemiological description. Consequently, we conducted an accurate analysis of hospitalization expenditure of the genitourinary system diseases to provide more data support and reference for the public health system.

In our study, we use the latest SHA2011 to calculate the elderly population health expenditures. The new health cost accounting system can accurately evaluate the actual burden of the aged, and the health expenditure of health institutions, diseases and people benefits from sharing dimension analysis [7]. In our analysis on the impact factors, because the huge health expenditure data does not obey the normal distribution, and ordinary multiple regression analysis cannot accurately describe the influence factors of hospitalization expenditure and the actual situation and cannot get reliable prediction effect and wide applicability. Therefore, we select the machine learning algorithm – neural network model to train the validation set to verify the model fitting degree of hospitalization expenditure of the genitourinary system diseases among the aged. Then we use sensitivity analysis to rank the various influencing factors and find the target points to draw the corresponding suggestions and measures of health policy.

## METHODS

### Data resources

Data were obtained from the 2015 Liaoning Health Statistical Yearbook, 2015 Liaoning Health Financial Yearbook, China National Health Accounts Report 2015, Liaoning Health Accounts Report 2015, etc. Demographic data were obtained from 2015 Liaoning statistical yearbook, medical institutions, and public health institutions.

### Study sample

We adopted multistage stratified cluster random sampling to investigate. The first stage was to select sample cities from Liaoning province, which based on the perfection of medical and health information system and the degree of economic development. Consequently, Dalian, Liaoyang, Panjin and Tieling was chosen as sample areas; the second stage was to select one county and one district randomly in each prefecture-level city and choose 3 community health service centers or township health centers in selected county and district and choose 3 villages in each county and then extract the 3 village clinics and clinics from them. After determining the sample areas and the sample size, the third stage was to fix a random sample according to the type of health agency and administrative levels including health care institutions and professional public health institutions.

Finally, seven provincial institutions, 58 institutions in Dalian, 62 institutions in Liaoyang, 64 institutions in Panjin and 61 institutions in Tieling were selected from the sample. The basic data information which collected from all patients in the hospital within one year consisted of age, gender, type of disease, type of medical institution, type of insurance, expenses, etc. The diagnosis of the diseases was coded according to the International Classification of Disease Tenth Revision (ICD-10).

The final valid sample size was 1 377 681 after excluding the invalid or wrong information. The influencing factor analysis of hospitalization care curative expenditure of 5993 cases for old patients with genitourinary system diseases whom we collected in Liaoning province was performed using neural network model and sensitivity analysis to rank the influencing factors of hospitalization curative care expenditure.

### Statistical method

#### Curative care expenditure accounted

Curative care expenditure (CCE) contained curative income1 and basic expenditure allowance2, which refer to outpatient and inpatient. The formula was shown as follows;


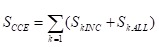
(1)

In the above formula, SkINC and SkALL represent the curative income and basic expenditure allowance respectively in different medical institutions. The formula of curative income was as follows:


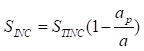
(2)


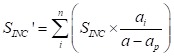
(3)

Above all of the formulas, curative income in per patient from the sample denoted as ai, total curative income denoted as a. Add all sample that contained preventive service on the basis of ICD-10, denoted as ap, then the sample’s curative income was aINC. Per patient sharing coefficient denoted by a_i_/(a – a_p_). Moreover, STINC represents the outpatient and inpatient’s income from 2015 Liaoning Health Statistical Yearbook and 2015 Liaoning Health Financial Yearbook. The purpose of formula (2) is to removed prevention expenditure of patients, and the formula of S_INC_ × a_i_/(a – a_p_) is to account the curative income in patient with the same age, gender, region, etc. The formula (3) means that could calculate curative income in various dimensions, such as age, disease, etc.

The formula of basic expenditure allowance was as follows:


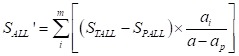
(4)

Those formulas about basic expenditure allowance were similar to curative income, the STINC, also collected from basic expenditure allowance from 2015 Liaoning Health Statistical Yearbook and 2015 Liaoning Health Financial Yearbook. The basic expenditure allowance in preventive service was collected from Yearbook too, denoted as SPALL. STALL – SPALL, means basic expenditure allowance.

The formula (4), split the basic expenditure allowance to per patient, which means the patient with the same age, gender, region, etc. in curative income, Curative income and basic expenditure allowance for old patient shared by above way. Mark off the different dimensions of age, gender, region, etc. for the elderly according to the result., and the total curative care expenditure in different age, sex, disease, etc., all could account from the above formulas (1).

#### Influencing factors analysis

Artificial neural network (ANN) model is a machine learning algorithm trained by back propagation (BP) algorithm [8]. Our study used the Multi-Layer Perceptron Regressor in library of Python Scikit-learn supported by Google to construct the regression model of hospitalization expenditure of the genitourinary system diseases for 65 years and over.

The main characteristics and advantages of the neural network model are mainly reflected in the following four points [9]:

1. The distribution storage of information and a certain extent of fault tolerance;
2. Parallel processing of information;
3. Self-learning and adaptive ability;
4. Nonlinear processing ability.

Our study used the hyperbolic tangent function as the active function and used second-order method L-BFGS as the optimization method for the loss function. 90% data was used as the training set. As for the validation set, we applied 5-fold cross validation strategy into the training set and 10% data was acted as test set. The loss function was the mean square error function with L2 regularization. The maximum number of training times was 1500.

The specific procedures were as follows:

**Step 1 – Data pretreatment.** The neural network model can analyze the non-normal distribution of health expenditure, but it cannot analyze the data set with missing records. Therefore, the data set containing missing errors should be eliminated first. Second, the health expenditure data are often unnormalized and with huge amount. The hidden layer of the neural network applied the nonlinear differentiable Sigmoid transfer function and used the gradient descent method in the study [10]. For example, for the logarithmic S function, if the argument is in the (0.1) range, the derivative function will become very small and the gradient becomes small. Then the decline and learning speed would be quite slowly, which may cause the network cannot converge [11]. Therefore, normalization of variables is one of the major tasks in data pretreatment. Our study applied the following formula to normalize: s_t_ = (X_i_ – mean)/std.

**Step 2 – Determination of network structure and training methods.** The complexity of the network is determined by the number of layers and the number of nodes in each layer. The neural network with simpler structure has faster convergence speed, but the accuracy of classification prediction is lower. The speed of neural network with more complex structure is slower, but the accuracy of classification prediction is higher. Consequently, the complexity of network structure, the accuracy of classification prediction and the efficiency of model training should be weighed [12]. The number of nodes in input and output layers is determined by actual problems. The complexity of the neural network is determined by the number of hidden layers and the number of nodes in hidden layers [11]. Our study followed the Occam’s razor principle and determined 5 nodes by using the trial and error methods in hidden layers. Training methods also affected the training results. The final sample size of our study was 5993, and used standard BP algorithm to update weights and applied L-BFGS algorithm as optimization method, which used two order information, had high precision and took up small memory.

**Step 3 – The determination of network weights.** Through the repeated analysis and study of the existing samples, the neural network can master the quantitative relations between the input and output variables and embody them in network weights. After determining the network structure, the core of neural network training is how to determine the network weights. The adjustment and corresponding calculation of network weights is an iterative process. When the error between the predicted result and the actual value of the output variable reached a smaller value, the determination of the network weight was a continuous iterative process.

#### Sensitivity analysis

In the 1990s, Garson and his colleagues proposed a sensitivity analysis method based on connection weights of the neural network which could explain the “black box” characteristics of neural networks [13]. The average error of the verification set should increase with the decrease in the number of iterations. The curve of fitting effect tends to be consistent with the increase in the number of iterations, and the average error is the same as the increase in the number of iterations [14]. With the number of iterations decreased, there is no over-fitting situation.

At the same time, we randomly selected 100 samples for verification and analysis. In the evaluation index of neural network, R2 and R2ADJ were used to reflect the degree of linear correlation between the independent variables and the dependent variables and cannot measure the degree of nonlinear correlation [15]. Consequently, we adopted the method and calculated the sensitivity index by 100, 500, 1000, and 2000 times, respectively, to sort out the rank of factors.

## RESULTS

### Fundamental result in curative care expenditure for the aged

In the distribution of curative care expenditure classified by the Global Burden of Disease (GBD), the highest proportion of CCE happened in noncommunicable diseases (NCD) (65%), followed by infections and maternal and child diseases, accounting for 15.65%; in the distribution of outpatient and inpatient expenditure, the inpatient expenditure of NCD accounted for 81.60%, which was the inpatient expenditure nearly four times (**Table 1**).

**Table 1:** The CCE classified by GBD I (%)

The name of diseases	Total expenditure		Out-patient	In-patient
Infections, maternal and prenatal diseases	15.65	18.58		15.08
Non-communicable diseases	65.96	55.72		70.28
Injury	8.56	3.90		10.12
Other (actual condition to reflect the illness and injury not classified)	9.83	21.80		6.45
Total	1	1		1

CCE – curative care expenditure, GBD – global burden of disease

In **Table 2**, it can be clearly seen that the proportion of outpatient and inpatient expenditure of NCD is higher than that of the other three diseases, and the hospitalization expenditure was 70.28%, which was the sum of the cost of hospitalization for the other three diseases more than twice.

**Table 2:** The CCE classified by GBD II (%)

The name of diseases	Total expenditure	Out-patient	In-patient
Infections, maternal and prenatal diseases	15.65	27.27	72.73
Non-communicable diseases	65.96	19.40	81.60
Injury	8.56	10.46	89.54
Other (actual condition to reflect the illness and injury not classified)	9.83	49.93	50.07
Total	1	-	-

CCE – curative care expenditure, GBD – global burden of disease

**Figure 1:**
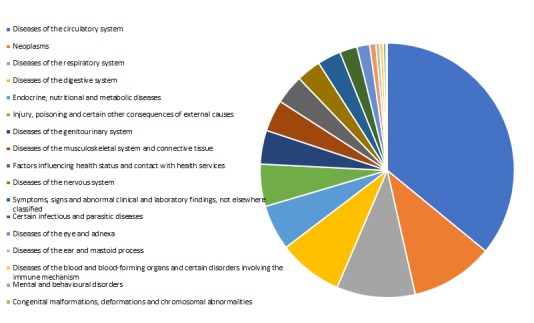
The curative care expenditure (CCE) of diseases classified by ICD-10 among patients aged 65 and over.

In the ICD-10 disease classification, the top three of the CCE for the elderly are circulatory disease, neoplasm and respiratory diseases. The proportion of the total incidence of genitourinary system diseases is not so high, but the diseases often caused by circulatory diseases complications such as severe chronic kidney disease, and some NCD can be regarded as the risk factors of genitourinary system diseases, as a result, we should pay more attention to the expenses of genitourinary diseases [16]. In the actual research process, the hospitalization expenditure of genitourinary system diseases was often ignored by researchers due to its low cost. For example, in 2014, 65 years of age and above in Liaoning province genitourinary system disease treatment costs 979.14 million yuan, ICD-10 coding disease accounted for only 4.29%. The cost of genitourinary disease is not significant compared to the overall cost (**Figure 1**).

### Allocation of medical institution for the aged in curative care expenditure

We divided the medical institutions into hospitals (general hospitals, Chinese medicine hospitals, specialist hospitals), primary health care institutions, public health institutions and emergency institutions. It is clear that most of curative care expenditure for the elderly was in general hospitals (184.16 million RMB), accounting for 92.96%, and the majority of the cost was in hospitalization expenditure (83.77%). In the subsequent analysis of the influencing factors, the data were mainly from it (**Table 3**).

**Table 3:** Distribution of outpatient and inpatient in different institutions

	CCE (billion RMB)	Outpatient (%)	Inpatient (%)
**Hospital:**	216.78	16.23	83.77
General hospital	184.16	12.92	87.08
Traditional Chinese medicine hospital	30.94	35.05	64.95
Special hospital	1.68	32.00	68.00
Basic medical institution*	15.53	63.67	36.33
Public health institution*	0.77	96.64	3.36
**Ambulatory facility**	0.10	100.00	0.00
Total	233.18	19.69	80.31
**The distribution of diseases of the genitourinary system in CCE (Million RMB)**	3349.89	631.75	2718.14

CCE – curative care expenditure, GBD – global burden of disease, RMB – renminbi

*Basic medical institution includes community health center and township health hospital; Public health institution includes disease control, maternity and child care specialist hospitals, etc.

### The CCE of diseases and genitourinary system diseases in different age

The whole population was divided into 0-14 years old group, 15-64 years old group, 65 years old and above, and we can find that per capita costs of 65 years old and above reached 4444.31 RMB combined with the Liaoning province population data, which is nearly four times compared with the other two groups. It demonstrated that we need to focus on the population of the burden of medical diseases and take measures specifically (**Table 4**).

**Table 4:** The CCE distribution of age group

Age group	Expenditure (billion RMB)	Proportion (%)	Number of population (thousand)	Per capita (RMB)
0-14	4.57	6.19	4469.10	1023.23
15-64	46.45	62.91	32 837.40	1414.61
>65	22.82	30.86	5135.50	4444.31

CCE – curative care expenditure, GBD – global burden of disease, RMB – renminbi

We extracted the genitourinary system diseases in the database and found that the hospitalization expenditure of genitourinary system diseases (2718.14 million RMB) accounted for 5.30% in the total hospitalization expenditure, outpatient costs accounted for 2.80%, 631.75 million RMB. From other perspectives, hospitalization expenditure of genitourinary system diseases accounted for 81.82% in curative care expenditure. In curative care expenditure distribution of the genitourinary system diseases in age groups, the average cost of people aged 65 years and over was highest (190.66 RMB) (**Table 5**).

**Table 5:** The CCE distribution of the genitourinary system by age group

Age group	Expenditure (million RMB)	Proportion (%)	Number of population (thousand)	Per capita (RMB)
0-14	35.67	0.78	4469.10	8.00
15-64	2322.61	5.00	32 837.40	70.73
>65	979.14	4.29	5135.50	190.66

CCE – curative care expenditure, GBD – global burden of disease, RMB – renminbi

### The evaluation of neural network model

**Figure 2:**
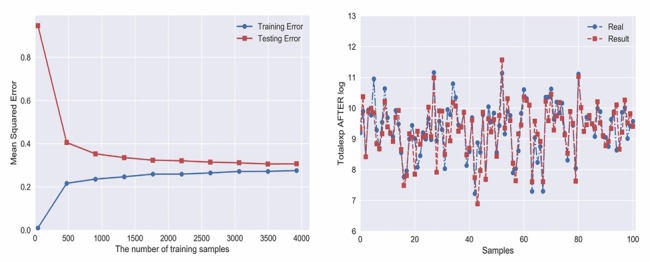
Fitting curve and learning curve of neural network model.

**Figure 3:**
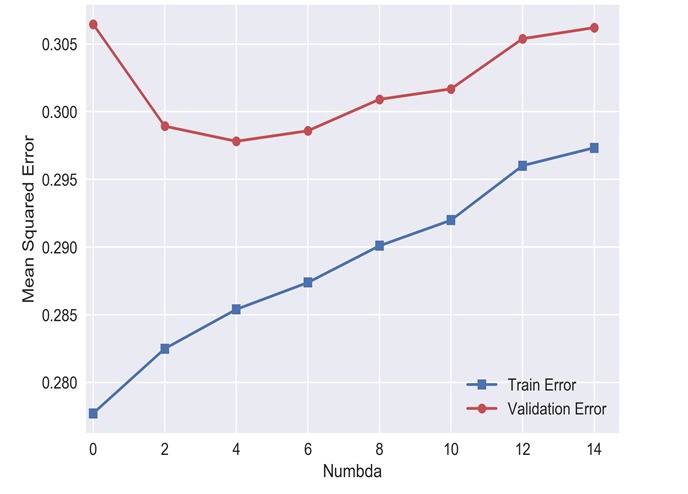
Cross validation curve of neural network model.

In the ANN evaluation, the errors are used to calculate the residuals, which can also reflect the degree of model fitting to some extent, especially when the sample size is large enough to be used for nonlinear model evaluation. In this study, R^2^ and adjusted R^2^ were 0.71 and 0.74, and MSE and MAE were 0.23 and 0.34, respectively, suggesting that the neural network model was better (**Figures 2 and 3**).

### Influencing factors of hospitalization expenditure by sensitivity analysis

In many studies, which focus on factors of hospitalization expenditure, length of stay, institutional level and type of institutions are the most common variables [15-17]. Based on those research facts, our research situation and existing data, we selected the type of insurances, admission season, gender and age as input layer variables, and hospitalization expenditure as output layer variables. The number of iterations was 100, 500, 1000 and 2000 times. The final results tend to be consistent, which can be considered the model convergence. In the importance of sorting, the length of stay (0.2826), the type of hospital institutions (0.1998), the type of insurances (0.1494) were the three top influencing factors (**Table 6**).

**Table 6:** Degree of sensitivity with different number of iterations

Number of iterations	Variables							
**Length of stay**	**Types of institutions**	**Types of insurances**	**Surgery or not**	**Gender**	**Levels of institutions**	**Age**	**Admission season**
100	0.2834	0.1978	0.1491	0.1179	0.1120	0.0970	0.0148	0.0280
500	0.2841	0.1986	0.1494	0.1214	0.1087	0.0955	0.0146	0.0277
1000	0.2830	0.1969	0.1499	0.1211	0.1109	0.0965	0.0138	0.0278
2000	0.2826	0.1998	0.1494	0.1207	0.1101	0.0955	0.0143	0.0277

## DISCUSSION

Chronic noncommunicable diseases have become the largest proportion of the disease burden of the elderly in China, and the incidence of diabetes in the world has risen sharply. The increasing number of young people suffering diabetes[16] and the economic burden led by these chronic noncommunicable diseases are what we should pay more attention to [17]. Diabetes will not only bring foot ulcers, retinopathy, but also bring cardiovascular disease, renal failure and other genitourinary system diseases and risk factors of genitourinary system diseases. The elderly who have been suffered from genitourinary system diseases usually had had the history of diabetes, which is likely to cause genitourinary infection, leading to the increasing economic burden of the disease for the elderly [18].

Our study found that the proportion of hospitalization costs for genitourinary system diseases was not the most prominent in comparison with other types of diseases by analyzing the curative care expenditure in Liaoning Province in 2014. According to Mccullough et al, most chronic noncommunicable diseases are risk factors and complications of genitourinary system diseases [19], especially chronic kidney disease, and the proportion of health resources and hospitalization expenditure consumed by these diseases are the highest part. By analyzing the hospitalization expenditure of genitourinary diseases, we can find some connections behind it and then control the costs of such diseases.

In the influencing factor analysis section, the ranking of the top three factors was the length of stay, the type of institutions and the type of insurances according to the degree of sensitivity. For the first two factors, the length of stay was the most important factor affecting hospitalization costs, which was often considered to be an indicator of medical resources consumption [20]. The longer length of stay, patients consumed more medical resources, and hospitalization expenditure was relatively higher. Domestic and foreign studies have shown that hospitalization expenditure has a positive correlation with the length of stay [21]. We suggest shortening the length of stay as an effective way to control hospitalization expenditure. In addition, which can increase bed rotation rate, and then limited resources can be served for more patients. It is another important role in strengthening and evaluating the hospital’s medical quality management.

On the other hand, the type of institutions has close relations with its level of diagnosis and treatment, size, technical accessibility and other related factors. However, this part of the agency’s investment costs finally would be added on the patient, aggravate the economic burdens and consume unnecessary medical resources [22]. Current health care situation in China – the overcrowding of tertiary hospitals but rare patients in primary medical institutions – is partly due to the imperfect health system and unclear orientation of health institutions [23]. Our results showed that the curative care expenditure in general hospitals accounted for the largest proportion, and the hospitalization expenditure accounted for the largest proportion of it, which indicated the current tendency of patients would like to choose the tertiary hospital as the first options, but the primary health care institutions failed to play role of the gatekeeper [24]. On the premises of ensuring the quality of medical care, it is reported that 65% patients in the tertiary hospitals in patients can choose to community health services for medical treatment, and 77% inpatients can refer to the primary health care institutions if primary health institutions work and rational allocation of medical and health resources are implemented well [25]. As a result, we should strengthen the construction of primary health care system and the primary health care system. Inspection and treatment equipment and other facilities are also essential. On the other hand, the primary health care services, medical technology, talent and other soft power construction should also serve as implemented.

We should put emphasis on improving the proportion of primary health care services in the medical service system, improve the utilization rate of health resources, optimize the stock of health resources, establish a scientific and orderly two-way referral system, and improve the classification of medical treatment and medical union construction. At the same time, to strengthen the gatekeeping role of primary medical and health institutions, the patient’s preoperative waiting and postoperative recovery into the primary hospital, is conducive to ease the tension at this stage of our relationship, promote the rational allocation of medical resources, control the general hospital the continuous consumption of health resources, thereby reducing the economic burden of patients. It will be never outdated that we focus on the improvement for quality of primary health care services in terms of reducing health expenditure.

The fundamental purpose of medical insurance is to share the economic risks of the disease to the individual and reflects the fairness [26]. Therefore, we should gradually reduce the different types of medical insurance patients to protect the gap between treatment and improve fairness. Wang Minghui and other studies have pointed out that the length of stay for medical insurance patients is longer than that of non-medical patients [27]. However, with the increase in the length of stay, the intensity of treatment is also declining. Consequently, we cannot draw a conclusion that the longer hospitalization brings better curative effect. Medical expenses are impacted by government, health care management, medical institutions, patients and other stakeholders [28]. The government should improve the relevant policies to strengthen the management of pharmaceutical and medical materials market, and reduce the layers of the increase in circulation to take a number of procurement programs. High-value consumables procurement should establish a unified price negotiation and supervision mechanism to eliminate the phenomenon of virtual high pricing [29], and a strict intervention system for access to surgery, regular organization of experts, comment on the hospital such medical behavior of the normative [30]. Medical insurance management departments should apply a variety of payment methods into the payment system. The medical insurance management department should adopt various payment methods combining the payment system and evaluate reasonable value timely according to the hospitalization expenditure of single diseases in medical institutions. For different types of insurances patients, the department should make targeted cost-control measures and play a full role of the third-party [31,32].

## CONCLUSION

In our study, hospitalization expenditure and influencing factors of 5993 cases of genitourinary system diseases among patients aged 65 years and over in Liaoning province were analyzed statistically. The results showed that the hospital expenditure accounted for the highest proportion of curative care expenditure. From the perspective of diseases distribution, the proportion of the expenses of genitourinary system diseases was not so high, but the average expenses of it among the patients aged 65 years and over were still in the highest, which is worth our thinking and attention. In the influencing factor analysis section, the length of stay, the type of institutions and the type of insurances were top three influencing factors. It suggested that the construction of hierarchical medical system made by the health sectors needs improvement. Health sectors should strengthen the gatekeeper role of primary health institutions and establish perfect hierarchical medical system to divert large hospitals patients to primary ones and encourage the establishment of medical union. We could implement various forms of methods to promote the downwards of high-quality health resources. In the insurance fund co-ordination, we should gradually eliminate the differences in current health systems in China, which is under three kinds of insurances. Health insurance department should concentrate on the establishment of a unified health insurance financing system. It is easy to manage and could eliminate the situation of selective medical treatment for patients.

## References

[1] Prince MJ, Wu F, Guo Y, Gutierrez Robledo LM, O’Donnell M, Sullivan R (2015). The burden of disease in older people and implications for health policy and practice.. Lancet.

[2] Zhang W, Shi W, Liu Z, Gu Y, Chen Q, Yuan W (2016). A nationwide cross-sectional survey on prevalence, management and pharmacoepidemiology patterns on hypertension in Chinese patients with chronic kidney disease.. Sci Rep.

[3] Wang Q, Brenner S, Kalmus O, Banda HT, De Allegri M (2016). The economic burden of chronic non-communicable diseases in rural Malawi: an observational study.. BMC Health Serv Res.

[4] Eckardt KU, Coresh J, Devuyst O, Johnson RJ, Kotgen A, Levey AS (2013). Evolving importance of kidney disease: from subspecialty to global health burden.. Lancet.

[5] Pateinakis P, Papagianni A (2011). Cardiorenal syndrome type 4-cardiovascular disease in patients with chronic kidney disease: epidemiology, pathogenesis, and management.. Int J Nephrol.

[6] Webster AC, Nagler EV, Morton RL, Masson P (2017). Chronic kidney disease.. Lancet.

[7] Zhang H, Wan Q, Chai P, Guo F, Zhai T, Wang C (2015). Estimation results of China curative care expenditure based on SHA 2011.. J Chinese Health Economics.

[8] Akerkar R, Sajja PS. Artificial neural network. Intelligent Techniques for Data Science. Berlin: Springer; 2016.

[9] Olden JD, Jackson DA (2002). Illuminating the “black box”: a randomization approach for understanding variable contributions in artificial neural networks.. Ecol Modell.

[10] Wang L, Zeng Y, Chen T (2015). Back propagation neural network with adaptive differential evolution algorithm for time series forecasting.. Expert Syst Appl.

[11] Graupe D. Principles of artificial neural networks. Singapore: World Scientific; 2007.

[12] Amato F, Lopez A, Pena-Medez EM, Vanhara P, Hampl A, Havel J (2013). Artificial neural networks in medical diagnosis.. J Appl Biomed.

[13] Maozhun S, Ji L, editors. Improved Garson algorithm based on neural network model. In: 29th Chinese Control and Decision Conference (CCDC), Chongqing, China 28-30 May, 2017.

[14] Valipour M, Banihabib ME, Behbahani SMR (2013). Comparison of the ARMA, ARIMA, and the autoregressive artificial neural network models in forecasting the monthly inflow of Dez dam reservoir.. J Hydrol (Amst).

[15] Vinodhini G, Chandrasekaran R (2016). A comparative performance evaluation of neural network based approach for sentiment classification of online reviews.. Journal of King Saud University-Computer and Information Sciences..

[16] Husdal R, Rosenblad A, Leksell J, Eliasson B, Jansson S, Jerden L (2017). Resource allocation and organisational features in Swedish primary diabetes care: Changes from 2006 to 2013.. Prim Care Diabetes.

[17] House AA, Ronco C (2011). The burden of cardiovascular risk in chronic kidney disease and dialysis patients (cardiorenal syndrome type 4).. Contrib Nephrol.

[18] Gallieni M, Aiello A, Tucci B, Sala V, Brahmochary Mandal SK, Doneda A (2014). The burden of hypertension and kidney disease in Northeast India: the Institute for Indian Mother and Child noncommunicable diseases project.. Scientific World Journal.

[19] McCullough K, Sharma P, Ali T, Khan I, Smith WC, MacLeod A (2012). Measuring the population burden of chronic kidney disease: a systematic literature review of the estimated prevalence of impaired kidney function.. Nephrol Dial Transplant.

[20] Lai C-I, Hung W-J, Lin L-P, Chien W-C, Lin J-D (2011). A retrospective population-based data analyses of inpatient care use and medical expenditure in people with intellectual disability co-occurring schizophrenia.. Res Dev Disabil.

[21] Nakamura K, Miura K, Nakagawa H, Okamura T, Okuda N, Nishimura K (2013). Treated and untreated hypertension, hospitalization, and medical expenditure: an epidemiological study in 314 622 beneficiaries of the medical insurance system in Japan.. J Hypertens.

[22] Lin W-Y, Chiu T-Y, Ho C-T, Davidson LE, Hsu H-S, Liu C-S (2014). Hospice shared-care saved medical expenditure and reduced the likelihood of intensive medical utilization among advanced cancer patients in Taiwan—a nationwide survey.. Support Care Cancer.

[23] Ren P, Xu Z, Liao H, Zeng X-J (2017). A thermodynamic method of intuitionistic fuzzy MCDM to assist the hierarchical medical system in China.. Inf Sci.

[24] Wang Y, Sun L, Hou J (2017). Hierarchical medical system based on big data and mobile internet: a new strategic choice in health care.. JMIR Med Inform.

[25] Li LW, Long Y. Health care experience of older persons with chronic illness in rural and urban China: a qualitative study in Shandong, China. 2016. Available: https://deepblue.lib.umich.edu/handle/2027.42/116798. Accessed: 15 September 2018.

[26] Wu J, Liu J, Zhu B, Mao Y (2016). Comparative analysis on the out-of-pocket expenditure among patients suffering from chronic kidney disease between medical insurance covered and uncovered areas in China.. Value Health.

[27] Hung W-J, Lin L-P, Wu C-L, Lin J-D (2011). Cost of hospitalization and length of stay in people with Down syndrome: Evidence from a national hospital discharge claims database.. Res Dev Disabil.

[28] Lavanchy D (2005). Worldwide epidemiology of HBV infection, disease burden, and vaccine prevention.. J Clin Virol.

[29] You CH, Kang S, Kwon YD, Choi JH (2013). Time trend of out-of-pocket expenditure among cancer inpatients: evidence from Korean tertiary hospitals.. Asian Pac J Cancer Prev.

[30] Duan W, Zheng A, Mu X, Li M, Liu C, Huang W (2017). How great is the medical burden of disease on the aged? Research based on “System of Health Account 2011”.. Health Qual Life Outcomes.

[31] Wang X, Sun Y, Mu X, Guan L, Li J (2015). How to improve the equity of health financial sources? Simulation and analysis of total health expenditure of one Chinese province on system dynamics.. Int J Equity Health.

[32] Yang Y, Zheng A, Li M, Duan W, Mu X, Wang X (2016). Medical economic burden of the ageing population: a multistage sampling analysis of 3 532 517 cases.. Lancet.

